# Characterization of Complex, Co-Adapted Skeletal Biomechanics Phenotypes: A Needed Paradigm Shift in the Genetics of Bone Structure and Function

**DOI:** 10.1007/s11914-014-0211-6

**Published:** 2014-04-23

**Authors:** L. M. Havill, H. B. Coan, M. C. Mahaney, D. P. Nicolella

**Affiliations:** 1 Genetics, Texas Biomedical Research Institute, P.O. Box 760549, San Antonio, TX 78245 USA; 2 Western Carolina University, Cullowhee, NC 28723 USA; 3 Southwest Research Institute, San Antonio, TX USA

**Keywords:** Bone, Osteoporosis, Genetics, Biomechanics, Bone fragility, Fracture risk, Composite traits, Bone mineral density, Co-adaptation, Fracture resistance, Mechanical load, Bone quality, Structural integrity, High-risk phenotypes, Phenotypic variation, Compensatory signaling, Gene pathways, Nonhuman primate, Baboon, Papio, Skeletal genetics, Phenotypic integration

## Abstract

The genetic architecture of skeletal biomechanical performance has tremendous potential to advance our knowledge of the biological mechanisms that drive variation in skeletal fragility and osteoporosis risk. Research using traditional approaches that focus on specific gene pathways is increasing our understanding of how and to what degree those pathways may affect population-level variation in fracture susceptibility, and shows that known pathways may affect bone fragility through unsuspected mechanisms. Non-traditional approaches that incorporate a new appreciation for the degree to which bone traits co-adapt to functional loading environments, using a wide variety of redundant compensatory mechanisms to meet both physiological and mechanical demands, represent a radical departure from the dominant reductionist paradigm and have the potential to rapidly advance our understanding of bone fragility and identification of new targets for therapeutic intervention.

## Introduction

Skeletal biomechanics are at the very heart of osteoporosis fracture risk. Susceptibility of a bone to fracture results directly from biomechanical properties that are maintained, in the face of changing, and sometimes competing, physiological and mechanical demands, by elegant co-adaptation of a myriad of traits at all of bone’s hierarchical levels of organization. Bone mineral density (BMD) was the initial focus of much bone biomechanics research aimed at understanding osteoporosis fracture risk at the exclusion of many other vital contributors to bone fragility, but great strides have since been made in identifying a vast number of morphologic and compositional bone properties that contribute to fracture resistance, and like BMD, are influenced by genetic variation. Understanding how, under which circumstances, and in whom this co-adaptation fails to maintain bone structural integrity under skeletal loading conditions that typically result in fracture is central to significant advances in osteoporosis prevention and treatment. Genetic studies of bone biomechanics designed to capture variability in the ways that co-adaptation of traits to habitual loading leads to bones that vary significantly in fracture resistance, hold the promise of leading us to the molecular biological mechanisms that drive the co-adaptation process itself, and that are likely to be central mediators of fracture susceptibility. Study designs that treat skeletal biomechanical phenotypes as multifactorial and reflect their true composite nature represent the beginnings of an exciting and necessary new paradigm in the genetics of skeletal fragility.

## Skeletal Biomechanics and Bone Fragility

Bone biomechanics broadly refers to the investigation of the material and mechanical properties of bone at multiple levels of hierarchical organization from the nano-scale level of the collagen and mineral composite, through the micro-structural organization of this composite into cortical and trabecular bone tissue, and the macro-structural organization of bone tissue into a functional organ. Bones are dynamic structures with the capacity to self-repair and to, within limits, alter material and shape properties to adapt to changes in habitual mechanical loading environments to which they are exposed.

The amount of bone in a region of interest (BMD) is a useful predictor of bone strength and of fracture risk [1] in clinical settings. This trait has been the subject of intensive osteoporosis research, but a great deal of osteoporosis fracture risk is independent of BMD [2, 3]. In fact, the structural integrity of bone, and, therefore, its resistance to failure, in any mechanical loading environment is an integrative function of a multitude of complex and interrelated characteristics of bone’s organic and inorganic matrix at the nano-scale, the organization of these constituents at the micro-scale, the spatial distribution of bone material, and the overall geometry of the bone at the macro-scale.

The adaptability of bone material and structure generally provides sufficient structural and material stiffness to support the loads of everyday life (functional loading). It has been postulated that, within certain constraints of minimal bone mass, it is maintenance of structural bone stiffness under the predominant functional loading directions (e.g., walking) that drives co-adaptation of bone traits to the predominant mechanical environment, rather than this co-adaptation being driven by a need to maintain structural integrity of the skeleton in a wide range of loading environments [4–6]. This functional adaptation may not be congruent with the ability to resist fracture in a fall, where the loads may be orthogonal to the dominant functional loading directions to which the skeletal structure is adapted. Thus, although large variations in the hierarchical organization or patterning of bone are able to provide adequate functional stiffness and strength for everyday activities, some of these patterns do not provide adequate strength during a nonhabitual loading event such as a fall.

## The Importance of Co-Adaptation of Traits to Bone Fracture Resistance

Ultimately, co-adaptation among all of bone’s traits, along with the direction and force of an applied load, determines whether or not a bone will fail (fracture) under particular mechanical loading conditions. Combinations of these traits can lead to maxima or minima in bone structural performance that cannot be reached by varying individual parameters alone. For example, it has been clearly shown in both rodent models [7–13] and, to a lesser extent, in humans [14–16], that co-adaptation of bone traits can lead to similar structural-level functional performance (e.g., slender vs. robust bones achieve similar bone structural stiffness with respect to body size via modifying tissue stiffness by increased tissue mineralization), and that genetic variation mediates the nature of this co-adaptation [8, 12]. Furthermore, although the co-adaptation of traits leads to bone structures that provide adequate function to counter daily loads, this process may produce bone structures that are variably suited to withstand atypical cyclic loads or adverse loading conditions such as a fall [9, 17].

## Complex Disease Genetics and Osteoporosis

The overarching goal of the search for genes that affect osteoporosis and bone fragility is to reveal the underlying molecular mechanisms that result in variation in disease risk or outcome in order to ultimately design treatments to prevent fractures in individuals. It is clear from the brief discussion of skeletal biomechanics above, that bone fragility has a heterogenous, pathogenetic, material and structural basis [3]. Risk of skeletal fracture is due not only to bony traits such as variation in bone density [18–23] and “bone quality”—a collective term comprised of bone geometry, porosity, architecture; physiochemical properties of the mineral, and organic phases; and accumulation of microdamage [24–28]—but also to impaired balance, reflexes, and muscle strength.

Genes that affect any one of these processes are potential candidates for contributing to the genetics of fracture risk. Add to this the complexities of age, sex, and skeletal site-specific genetic effects; gene-gene interaction; and gene-environment interaction, and it is no wonder that identification of genes that largely explain population-level variation in bone fragility and fracture risk is a slow and tedious process. It is also not surprising that the intricacies of the functional effects of genes and gene variants associated with osteoporosis, and the magnitude of their importance to population-level disease risk, are rarely understood.

## Recent Gene-Specific Progress

Although it is abundantly clear that many genes contribute to variation in skeletal biomechanics and, by extension, fracture risk, focused investigations of single gene pathways have advanced our understanding of skeletal biomechanics. These studies can be broadly grouped into categories that describe the genetic underpinnings of specific biological traits. Studies that investigate the genetics of cell/tissue response to mechanical and environmental stimuli are extremely common. These use mouse and cell culture models to investigate bone tissue- and cell-level bone response to such stimuli as mechanical load, inflammation, and dietary intake. The use of mouse and cell culture models allows for isolation and testing of very specific stimuli. These studies reveal some promising new results. One such result links the calcineurin/NFAT pathway to inflammation and osteoclastogenesis [29]. A recent study in cultured cells shows that mechanical stimulation of individual cells potentiates calcineurin/NFAT signaling and stimulates osteoblastogenesis [30]. New research also suggests a link between orthopedic particle-stimulated osteoclastogenesis and NFAT signaling [31•]. Orthopedic implants have been shown to release particles that initiate a host immune response, leading to osteoclastogenesis at the bone-implant interface. The inflammatory NFAT pathway was shown to play a role in the resulting osteoclast-mediated bone resorption. These studies provide support for a firm link between inflammation and bone fragility. This pathway may represent an important new direction in the study of bone fragility that has previously received little attention with regard to fracture risk. The link between inflammation and bone fragility/fracture healing is also important in studies that investigate compromised bone quality in diabetes and metabolic-related syndrome [32•, 33].

Another interesting development involves a novel high throughput mouse model in the assessment of bone mechanical traits in combination with quantitative trait loci (QTL) analyses [34•]. This study illustrates the importance of experimental manipulation and control that are possible in mouse and cell culture models. Although this study is a first pass for identification of QTLs for specific traits of interest, the study design has broader implications for using high throughput methods to assess a wide range of traits that we know to contribute synergistically to fracture resistance. Inbred mouse models, as opposed to outbred models that are more genetically similar to humans, are amenable to this study design because the model organism can be manipulated one gene at a time to test individual stressors.

Mouse and cell culture models have also been valuable to investigation of the genetics of bone structure and quality. Studies focused on bone’s organic extracellular matrix have become increasingly important as the field has moved beyond a BMD-centric view of fracture resistance. A wide variety of studies in this area are leading to new insights into the role of the extracellular matrix (ECM) and matrix-cell or matrix-matrix interactions in bone quality. Studies in this area include the role of bone matrix transcription factors in bone fragility. Specifically, ATF4, a transcription factor responsible for bone ECM deposition, has been implicated in bone toughness in inbred mice [35•]. Also, work in a mouse model of osteogenesis imperfecta indicates that genetic mediation of ECM connections and formation may illuminate the role of bone’s organic matrix in fracture risk [36].

Other gene-specific studies are designed to investigate the genetics of basic bone homeostasis. The research in this area is extensive and the genes and pathways that potentially impact bone fragility are numerous. There are obvious pathways, however, for which there is strong support linking them to bone fragility variation. For example, canonical Wnt signaling is well known for its involvement in bone formation and resorption. New findings indicate that Wnt signaling controls bone resorption through more than one pathway, and likely, in response to various stimuli [37]. Ongoing research into the role of Wnt signaling will undoubtedly provide additional critical insight into the role of this pathway in bone homeostasis and adaptation to mechanical loading. Another important area of focus involves the regulation of osteoblast/osteoclast activity (RANKL/OPG signaling) [38]. In fact, a recent review highlighted the importance of using and developing mouse models to study this particular pathway [39•].

These studies are a sampling of a multitude of single gene/pathway studies aimed at teasing function out of single genes within the myriad of bone signaling pathways. Some of these studies undoubtedly will provide valuable insight into the mode of action of potential fracture risk genes. The limitations of these studies, however, are that they cannot address how the pathways interact with each other or with other important pathways to produce normal variation in bone fragility, nor do they give us insight into the relative importance of each pathway in determining population-level fracture risk variation.

## Bone Structural Integrity and Identification of High Risk Phenotypes

As skeletal biomechanics research initially focused on easily measureable and clinically implemented BMD, the genetics of skeletal biomechanics followed suit, focusing strongly on genes that affect variation in BMD. We now know that the structural integrity of bone results from variation in the co-adaptation of a multitude of complex and interrelated bone traits, and that co-adaptation of these traits provides for redundant combinations of traits that can result in bones that are functionally adequate under routine loading conditions [7, 15, 17]. These combinations can involve quite different sets of bone traits, a subset of which are suboptimal when subjected to atypical or traumatic loads, resulting in bones (and individuals) that are more likely to sustain fracture. Identifying these suboptimal trait combinations, however, is extremely challenging. To do so we must simultaneously account for variation at each of bone’s hierarchical levels of organization and move beyond a uni-dimensional treatment of the relationship between bone characteristics and bone performance. Many of the bone quality measurements now known to be important components of bone fragility can only be measured directly from a bone sample, which is much too invasive to be realistic on a large scale, or at the mechanically and clinically most relevant skeletal sites. This has placed severe limitations on our ability to incorporate these measures into our genetic analyses.

## Paradigm Shift: Phenotype Integration

The dominant study design in skeletal genetics and bone biomechanics focuses on the role of 1 or a limited set of morphologic and/or compositional factors in bone fragility.

Because choice of phenotype necessarily limits the genes that can be discovered (as noted in an excellent review of genomics studies of osteoporosis through 2009) [40], this focus on a single or reduced set of bone traits limits our ability to discover genes that have much broader, overarching effects on bone trait co-adaptation. This reductionist approach was both necessary and productive, as we first began to appreciate the degree to which BMD variation left fracture risk unexplained, then set out to identify other bone qualities that affect bone’s structural integrity, and then the extent to which such traits were genetically mediated. Continued adherence to this reductionist paradigm will not, however, reveal a complete picture of the mechanobiological processes underlying bone fragility.

Because co-adaptation of a large suite of bone traits underlies fracture risk, osteoporosis risk research requires a more conceptually broad, holistic study design that captures variation at each of bone’s hierarchical levels, because all of these traits work synergistically to control fracture risk. An approach that is likely to be more productive, but is difficult to achieve, is one in which phenotypes are multifactorial themselves, capturing variation in many bone traits at once, thereby reflecting the complex interactions among bone traits that ultimately lead to bone fragility.

## The New Paradigm

Some research groups are applying approaches that represent this new paradigm of phenotypic integration and, in so doing, are (1) addressing gaps in our knowledge as to how complex bone traits work in concert to confer robust bone strength and fracture resistance, and (2) investigating the genetics of skeletal biomechanics in a way that is likely to quickly and significantly advance the field, and, ideally, our ability to develop focused treatments that target specific deficits that render individuals (or sets of individuals) more susceptible to osteoporotic fracture. These studies lead us in an exciting new direction with the potential to vastly improve our fundamental understanding of the intricacies of the skeleton’s ability to maintain structural integrity in the face of diverse and changing mechanical and physiological demands. With inbred rodent studies revealing that genes likely mediate the course of co-adaptation of traits in response to the skeletal environment [8, 12], these studies have the potential, also, to lead us to quantitative phenotypes that more directly reflect the co-adaptation processes in which we are most interested, and the genes that regulate them.

In a series of manuscripts Saless et al. [41•, 42–44] report the results of using a comprehensive phenotyping approach and a reciprocal intercross of HcB-8 and HcB-23 mouse strains to map and identify genes that account for variation in bone biomechanical performance, BMD, bone geometry, and tissue-level properties. These authors [42] identified pleiotropic QTL affecting multiple phenotypes, including an especially robust QTL on mouse chromosome 4 that, after normalization for body size, persisted for whole bone biomechanical performance, many shape parameters, and BMD. They also assessed and analyzed femoral material properties [43], achieving similar results, and gaining insight into the nature of the previously located chromosome 4 QTL through its absence in the well-powered material properties analysis (i.e., the QTL appears to represent a gene that acts via modeling rather than through variation in material properties). These studies further illustrate the advantages of comprehensive phenotyping and simultaneous analysis when, through the use of variable reduction methods (i.e., principal components analysis (PCA)) they identify a QTL that was not detectible via examination of any of the traits individually [41•]. The occurrence of QTL that affect multiple traits, and the ability of PCA to reveal genetic signals that are not detectible in the absence of the shared information among multiple phenotypes, demonstrates that exploiting the complexity of the co-adaptation of bone traits to maintain structural integrity is a promising avenue for identifying the genes and associated biological processes involved in osteoporosis risk.

In a study of recombinant mice, Jepsen et al. used a different approach of phenotypic integration in which they mapped QTLs for skeletal robustness, morphologic compensation, and tissue mineralization—all components of a phenotypic covariation network of compensatory bone trait co-adaptation to maintain functional loading integrity [45]. Their results suggest, not surprisingly, that individuals with slender bones will not necessarily be genetically predisposed to weak bones unless the individuals also have a genetically mediated deficiency in morphologic compensation (the ability to, through other heritable traits, compensate for the slenderness of their bones). This group focuses their work strongly on the relationships among bone robustness, relative cortical area, and stiffness [46•, 47, 48•, 49•], but is using covariance among these traits to gain important insight into bone’s capacity for co-adaptation of traits at different levels of its hierarchical organization to maintain fracture resistance. Although they are able to demonstrate genetic regulation of co-adaptation of traits in rodents, their analyses of the relationships among these traits in human tibiae and femoral neck begin to address the question of to what degree, and in what manner, the rodent results translate to population-level variation in humans. In a review of current knowledge, Jepsen notes that the biologic mechanisms that coordinate the network of adaptive responses and compensatory traits are not well understood [50].

The research of our own group with bones from a unique population of genetically characterized pedigreed baboons (*Papio hamadryas* ssp.) involves a thorough characterization of a wide array of bone traits known to be associated with bone fragility. Vertebral bone mechanical properties [51•], cortical bone mechanical properties [52], intracortical remodeling of the femur [53], and femoral bone shape [54] are strongly heritable in these baboons. The results of our study of the biomechanical performance of vertebral trabecular bone reveals independent genetic effects on toughness that are not accounted for by BMD [51•]. This nonhuman primate model is a superior animal model to complement more widely available rodents, as it is genetically and, consequently, physiologically and anatomically very similar to humans with regard to skeletal aging specifically and complex metabolism in general [40, 55]. We use advanced statistical techniques to distill this information into manageable independent composite traits that we then relate to experimentally determined biomechanical testing outcomes to elucidate the complex relationships of these combinations of traits in their contribution to fracture resistance. In addition, because these femurs are from members of a single large, extended pedigree, we are able to investigate the degree to which genetic variation among pedigree members of this outbred population is responsible for variation in the composite traits (i.e., estimate heritability of the composite descriptors of variation in bone traits). Table 1 lists the extensive list of phenotypes we are including in this analysis, which we believe to be the most comprehensive set yet attempted.

**Table 1:** Traits included in comprehensive phenotyping of bones of pedigreed baboons

Trait group	Individual trait
Neck histomorphometry and geometry	Osteon area Haversian canal area Percent osteonal bone Osteon population density Percent void space Cross-sectional area Cross-sectional moment of inertia Minimum principal moment of inertia Maximum principal moment of inertia Polar moment of inertia
Collagen content crosslink characterization	Hydroxylysyl-pyridinoline Lysylpyridinoline Pentosidine
Microdamage Crack length	Crack number Crack density Crack surface density
Midshaft geometry	Cross-sectional area Cross-sectional moment of inertia Minimum principal moment of inertia Maximum principal moment of inertia Polar moment of inertia
Midshaft histomorphometry	Osteon area Haversian canal area Percent osteonal bone Osteon population density
Physicochemical properties	Crystallinity Carbonate substitution Mineralization Collagen crosslinks (mature/immature)
Matrix material properties	Tissue elastic modulus
Porosity	Bone porosity Pore size distribution Mobile water Bound water
Trabecular bone Architecture	Bone volume fraction Connectivity density Trabecular number Trabecular thickness Trabecular separation Degree of anisotropy
Ash content	Apparent density Tissue density Tissue volume fraction Percent mineralization
Hip structural analysis	Femoral neck length Neck-shaft angle Hip axis length Calculated for narrow neck, intertrochanteric, and shaft: Areal bone mineral density Cross-sectional area of cortical bone Cross-sectional moment of inertia Subperiosteal width Estimated endosteal diameter Estimated average cortical thickness Profile center distance Center of mass position Section modulus Buckling ratio

## Conclusions

Although challenging, genetics of skeletal biomechanics is a critical area of osteoporosis research. Identification of the genes that underlie variation in skeletal biomechanics will lead us to the biological mechanisms that result in greater or lesser osteoporosis fracture susceptibility. At present intervention still centers on altering an individual’s BMD. A more holistic treatment of skeletal biomechanics and strength is necessary to move the field toward development of effective means for identifying or treating individuals who are at high risk for fracture, but that are of normal BMD. It may also give us the knowledge to, eventually; understand co-adaption processes that result in individuals who, in spite of low BMD, do not have fragile bones. It is likely that achieving these goals will require capitalizing on the strengths of various animal models, including the more easily manipulated rodents, to the more closely related and, hence, more metabolically and genetically similar nonhuman primates.
